# Kinematics and kinetics of dogs walking over increasing heights of cavaletti exercise

**DOI:** 10.1016/j.heliyon.2024.e40952

**Published:** 2024-12-04

**Authors:** Katie Clarke, Jade Terry, Scott Blake, Roberta Ferro de Godoy

**Affiliations:** Anglia Ruskin University, Writtle School of Agriculture, Animal and Environmental Sciences, Lordship Road, Chelmsford, CM1 3RR, United Kingdom

**Keywords:** Ground reaction forces, Pole, Remedial exercise, ROM

## Abstract

The purpose of this study was to identify the effects of four heights of cavaletti exercise on canine kinematics and kinetics. Eight dogs had kinematics and kinetics data collected whilst completing cavaletti pole exercises at four different heights (floor, mid-metatarsal, hock, and stifle). Dogs had anatomical markers placed on bony landmarks of the fore and hindlimb joints. Each trial was recorded via a high-speed camera and two pressure mats. Kinematics outcomes were joint range of motion (ROM). Kinetic outcomes were force (%BW), impulse and peak pressure. Friedman and Wilcoxon's tests were performed to identify significant differences between cavaletti heights. A statistically significant increase was found at hock height placement across all joints whilst maintaining kinetic parameters similar to floor height. No significant differences were found for hip range of motion at any pole height. It was found that stifle height showed no increase in joint range of motion but has the highest forces involved. This determines that hock height is the most beneficial height for improving joint range of motion whilst not increasing forces upon the limbs. Stifle height should be avoided, in some conditions, due to high peak pressures, which do not necessarily lead to a significant further increase in range of motion.

## Highlights

• Cavaletti poles at hock level is sufficient to increase ROM of all joints, except hip.
• Cavaletti poles up to hock level does not significantly increases forelimb loading and limbs peak pressures.
• Cavaletti poles at stifle height increases forces and peak pressures without additional benefits in kinematics.

## 1 Introduction

It was not until recently that physical rehabilitation became an accepted part of comprehensive veterinary medicine and surgery [1]. Veterinary physiotherapy is believed to be a continuous process [2], that aims to optimise recovery, enhance performance and minimise dysfunction in patients with orthopaedic conditions [3]. Musculoskeletal rehabilitation aims to restore limb range of motion and function by enhancing the flexibility of soft tissues and strengthening of musculature. This is expected to improve the joint biomechanics and functional mobility of the patient [4,5].

Many treatment options are available such as manual therapy, electrotherapies and therapeutic exercise [6]. Such exercises heighten proprioception, balance, muscular strength, and range of motion (ROM) and re-educate the gait [7]. Cavaletti poles are often utilised to challenge patients by maximising joint angles and ensuring they stabilise themselves whilst performing the exercise [8]. Progression can be made by raising cavaletti poles to certain heights, to suit the canine characteristics of length and height [9]. Cavaletti poles are suggested as low impact and therefore incorporated into rehabilitation plans for orthopaedic conditions [5]. A recent study has studied basic temporospatial kinematics and kinetics of walking over different heights of cavaletti and has used a similar pressure-sensing system for the ground reaction forces [10]. Cavaletti height did influence particularly velocity, but there were no conclusive results about the ground reaction forces. Therefore, studies are limited in identifying the effects of supposed low-impact exercises on the loading and joint motion as the cavaletti height increases.

It is yet to be investigated how different heights of cavaletti poles affect joint range of motion and ground reaction forces upon the limbs when landing. Acknowledging such effects is vital for veterinary physiotherapists in order to not exacerbate orthopaedic conditions that need maintenance or low-impact exercise.

As for most of the rehabilitative exercises for dogs, there is a limited information on cavaletti poles and this is the first one to report both joints range of motion (ROM) and kinetics aspects simultaneously whilst exploring different heights of the exercise. The aims of the study were to analyse the kinetics and kinematics of dogs walking over four heights of cavaletti poles. For a practical application, we wanted to identify the optimal cavaletti pole height for achieving increased joint range of motion, whilst not significantly increasing ground reaction forces exerted during walking over various cavaletti pole heights. To obtain the required data peak vertical forces were measured simultaneously with joint angle kinematics. We hypothesised that by increasing cavaletti pole height we would increase joint ROM and also ground reaction forces.

### 1.1 Materials and methods

A randomised cross-over design was used to investigate the effect of cavaletti poles at varying heights on canine joint range of motion (ROM) and kinetics, during a walking gait.

#### 1.1.1 Ethical approval

The data were acquired according to modern ethical standards and was approved by the Animal Welfare and Ethics Committee of Writtle University College. The approval number is 98354129/2018. Written informed consent was obtained from the owners of the participants of the study. Veterinary consent was also required to discount any current or underlying orthopaedic conditions that could hinder results.

#### 1.1.2 Animals

Eight dogs (n = 8) were used in this experiment, five females and two males of various breeds and sizes, representing the diversity of the canine species (Table 1). To be included in the study, dogs were required to have veterinary permission to ensure they had no pre-existing conditions. The sample size was calculated using the resource equation approach [11] and also based on published studies assessing effects of cavaletti poles exercise on canine kinetics [8]and kinematics [12]with both studies achieving adequate statistical discrimination with 8 dogs. Assuming the methods differ slightly in their intervention and outcomes, estimates by 10 %, for Type I and II errors of 0.05 and 0.20, respectively, using the Bland-Altman Test we estimated a sample size of between 5 and 11 dogs would be needed, therefore the decision to use 8 dogs on this study (MedCalc® Statistical Software version 20.115).

**Table 1:** Gender, age, breed, and weight (kg) of the selected participating canines.

Subject No.	Breed	Gender	Age	Weight (kg)
1	Bodeguero	Female	1.5	9.55
2	Cocker Spaniel	Female	4	12.05
3	Boarder Terrier	Male	7	9.1
4	Parsons Terrier	Female	6	6.9
5	Huskey x Labrador	Male	3	21.55
6	Pug	Female	7	6.15
7	Boarder Terrier	Female	7	8.1
8	Pug	Female	5	5.25

Veterinary consent was also required to confirm absence of any current or underlying orthopaedic conditions that could hinder results.

#### 1.1.3 Experimental design

Dogs participating in the trial were warmed up with 5 min of walking on leash, then asked to perform each one of the four heights of the pole exercises, in random order and were required to perform the exercise five times, with the three middle runs being used to analysis (first and last run discarded). This disregarded the chances of a gradual warm-up and reduced habituation as the heights were adjusted. It is well reported that active warm-up can increase joint ROM and performance [13] supporting randomisation of the order in which dogs walked over the various heights of cavaletti poles.

#### 1.1.4 Markers placement

**Fig. 1:**
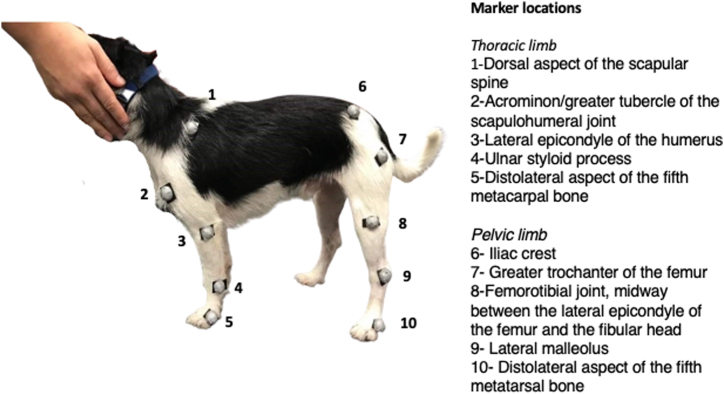
Markers were placed on the skin with double-sided adhesive tape at anatomical landmarks.

To ensure the reliability of joint angles measurement, 19 mm spherical reflective markers were placed on the joint centre of rotation. Placement of the markers on the forelimb were: dorsal border of scapula, humeral greater tubercle, humeral lateral epicondyle, ulnar styloid process and lateral aspect of the fifth metacarpal bone. Hindlimb markers were placed on the; iliac crest, femoral greater trochanter, femoral lateral epicondyle, lateral malleolus and lateral aspect of the fifth metatarsal bone (Fig. 1) [14,15].

Markers were used to identify joint angles at maximal flexion (⁰) and maximal extension (⁰), as previously described [12,16]. The markers were attached to each dogs’ skin, using double-sided tape, by the same experienced researcher to ensure repeatability.

#### 1.1.5 Cavaletti exercise

Dogs were required to walk over a sequence of five cavaletti poles for the exercises. For each sequence, poles were placed at four heights: floor, mid-metatarsal level, hock level and stifle level. Floor height was the lowest pole setting due to being an introductory height and this allows progression to be made in real-life situations.

**Fig. 2:**
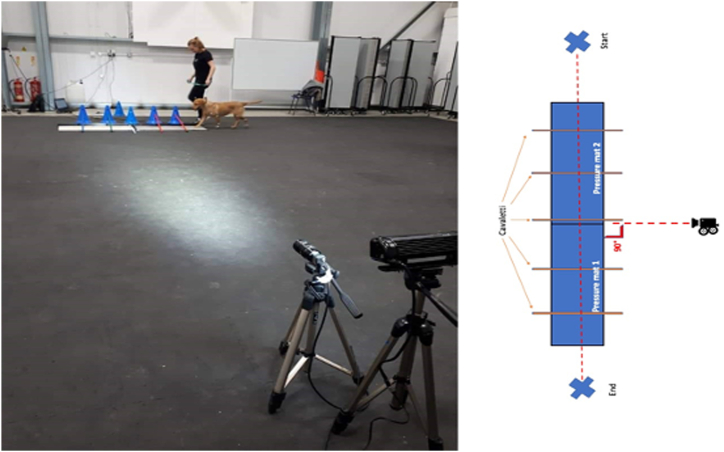
Experimental setup: Equidistant cavaletti were adjusted to enable recording of each limb walking over two pressure mats. High-speed camera was positioned perpendicular to the dog walkway.

Cavaletti distance altered depending on the subject's size and length enabling each dog to comfortably walk over the poles whilst only placing one limb at a time between each. Measurements were taken from the elbow joint to the floor, to assist with appropriate pole spacing. The poles' heights depended on where the specific joint was on the dog. Cavaletti were positioned over two pressure mats and 6m away and perpendicular to the high-speed camera (Fig. 2).

#### 1.1.6 Kinetics data collection

Kinetic gait parameters were collected by walking dogs over two commercially available pressure-sensing walkway mats (7101HL, Tekscan, Norwood, Massachusetts, USA) placed sequentially in a longitudinal way. Each one of the walkways consisted of two sensors mounted on a rigid platform, with an overall size of 148.5 × 58.4 cm and sensing area of 146.3 × 44.7 cm. The mat contained 4 sensel per cm^2^. The mat was 0.6 cm thick and collected at 185Hz, allowing kinetic data to be analysed using proprietary software (Tekscan Walkway, v7.02, Norwood, Massachusetts, USA). The walkway mats were calibrated according to the manufacturer's instructions using a step calibration and the body mass of one of the researchers, a real-time check was then performed by walking across the walkway to ensure consistency in the amplitudes and duration of the forces. The walkway mats were used to record the kinetics data for individual limbs for the duration of the cavaletti exercise. Kinetic data were recorded in synchronisation with kinematic data and video recordings. Dogs were walked over the walkway three times or until three valid recordings were gathered. A valid run was considered to be a dog walking across the mat at a constant speed without hesitating, stopping, or trotting.

#### 1.1.7 Video collection

A high-speed camera (Quintic USB3 1.3 MPixel High-Speed Camera, Quintic Consultancy Ltd., Birmingham, UK) which captured data at 300Hz was used to collect videos. The camera was positioned on a tripod perpendicular to the pressure walkway mats to allow for the collection of all the cavaletti exercises. Markers were illuminated by a 5W halogen lamp positioned above and behind the high-speed camera. The subjects were walked past the capture window until three successful cycles were captured. A valid cycle consisted of a controlled walk with the dog walking straight, with all limbs passing over the cavaletti poles individually and the pressure mats [17].

To validate clinical findings and eliminate handler influence, the same experienced handler was used to walk the subjects on each trial. The dog collar and lead were attached to a tension gauge (Telerein C IT, New Zealand), which gave real-time indications of the force transmitting down the lead. A limit of 5 N (N) of tension was acceptable through the trial verifying that pressures used were standardised throughout.

#### 1.1.8 Data analysis

A motion analysis software (Quintic Biomechanics, Quintic Consultancy Ltd, Birmingham, UK) was used to complete video analysis. As subjects passed the camera window, the stride that was most central to the camera was selected with the footfall of the forelimb being placed before the flight arc commenced over the poles. A full stride counted as the craniocaudal distance between the initiation of the stance phase and the conclusion of the swing phase for a given forelimb paw. This allowed automatic digitisation to track the joint angles of the bony landmarks over an entire gait sequence. For each parameter analysed a minimum of three trials were pooled for each dog to create a representative which allowed dynamic ROM angles to be calculated from the maximum flexion and maximum extension angles for each single joint within a stride.

Kinetic data was analysed by dedicated software (Tekscan Walkway, v7.02, Norwood, Massachusetts, USA). Kinetic parameters consisted of individual limb force (%BW), impulse and peak pressure.

A minimum of three strides for each dog was analysed for kinematics and kinetics and averaged to create a representative.

#### 1.1.9 Statistical analysis

Once all outcomes were analysed, data was transferred to SPSS v26.0. The mean ± SD of the outcomes variables at each cavaletti pole height t was calculated and subjected to a Shapiro-Wilk test for normality which showed non-parametric data. Following this, data underwent assessment by Friedman's test. Post-hoc analysis was then conducted with Bonferroni correction (95 % confidence interval (CI)) assessments in order to determine where the statistically significant differences between cavaletti heights lay. The results report SPSS Bonferroni adjusted p-values. Furthermore, Wilcoxon signed-rank tests were used to identify any differences between forelimb and hindlimb kinetics. The significance level was set at 95 % CI (p < 0.05). The results report SPSS Bonferroni adjusted p-values.

## 2 Results

All dogs completed successfully the trial. Data are median (*Mdn*).

### 2.1 Kinematics

#### 2.1.1 Shoulder ROM

**Fig. 3:**
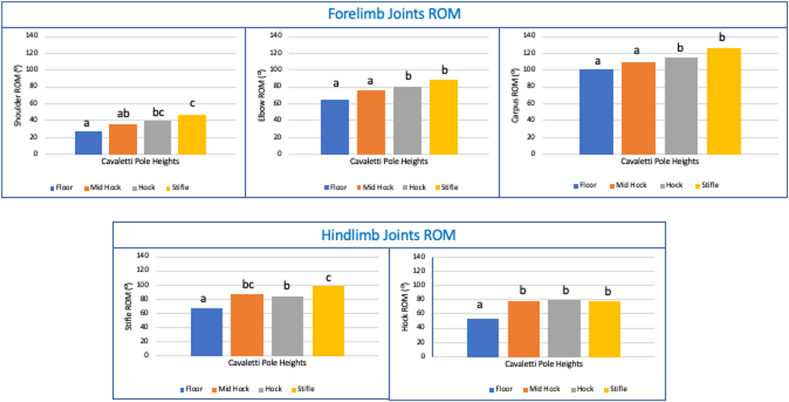
Median scores of forelimb and hindlimbs joints range of motion (ROM) in dogs (n = 8) walking over different poles heights. Different letters indicate significant differences p < 0.05 by Friedman's test.

Shoulder ROM was significantly different depending on cavaletti heights (χ^2^(3) = 27.6, p < 0.0005). A post-hoc analysis shown a substantial increase in shoulder ROM when poles are raised from height (*Mdn* = 26.62°) to hock height (*Mdn* = 39.56°) (*p* = 0.001) and from mid-metatarsal (*Mdn* = 35.66°) to stifle height (*Mdn* = 46.42°) (*p* = 0.037) (Fig. 3).

#### 2.1.2 Elbow ROM

Elbow ROM was statistically significantly different at the different pole heights (χ^2^(3) = 22.275, p < 0.0005). Post-hoc analysis revealed statistically significant increase in shoulder ROM when poles are raised from floor height (*Mdn* = 65.5°) to both hock height (*Mdn* = 80.6°) (*p* = 0.006) and stifle height (*Mdn* = 88.76°) (*p* < 0.0005) (Fig. 3).

#### 2.1.3 Carpus ROM

There was a significant difference in carpal ROM between the different cavaletti heights (χ^2^(3) = 13.95, p < 0.003). Pairwise comparisons identified increases that were significant differences in carpus ROM when poles were raised from floor height (*Mdn* = 100.94°) to stifle height (*Mdn* = 125.71°) (*p* = 0.037) and raised between mid-metatarsal (*Mdn* = 109.21°) (*p* = 0.002) and stifle height (*Mdn* = 125.71°) (Fig. 3).

#### 2.1.4 Hip ROM

Hip ROM was not statistically significant different with the different cavaletti heights (χ^2^(3) = 2.7, p = 0.44).

#### 2.1.5 Stifle ROM

Stifle ROM was statistically significantly different at various cavaletti heights in comparison to ground level (χ^2^(3) = 32.475, p < 0.0005). Post-hoc analysis indicated a significant increase in stifle ROM between poles at floor height (*Mdn* = 68.16°) to those at mid-metatarsal height (*Mdn* = 87.47°) (*p* = 0.004) as well as between poles at hock height (*Mdn* = 84.64°) (*p* = 0.002) to stifle height (*Mdn* = 98.69°) (*p* < 0.0005) (Fig. 3).

#### 2.1.6 Hock ROM

Comparisons of the four cavaletti heights were performed to identify differences in hock ROM using Friedman's test, which showed a statistically significant difference (χ^2^(3) = 19.875, p < 0.0005). Post-hoc tests showed significant increases when poles were raised from floor height (*Mdn* = 52.62°) to hock height (*Mdn* = 79.18°) (*p* = 0.006) as well as from floor heigh to stifle height (*Mdn* = 78.23°) (*p* < 0.0005) (Fig. 3)

#### 2.1.7 Percentage of increase in joint ROM

Table 2 shows the individual percentage (%) increases of joint angles from floor height.

**Table 2:** Percentage increases of shoulder ROM in dogs (n = 8) during walking over cavaletti pole heights, in comparison with ground cavaletti.

Joint Angle	Mid-metatarsal%	Hock%	Stifle%
**Shoulder ROM**	30.38 %	42.55 %	65.70 %
**Elbow ROM**	12.98 %	18.39 %	39.57 %
**Carpus ROM**	−16.81 %	5.22 %	14.99 %
**Hip ROM**	14.99 %	30.81 %	23.07 %
**Stifle ROM**	26.10 %	31.94 %	47.12 %
**Hock ROM**	37.75 %	53.30 %	56.53 %

#### 2.1.8 Kinetics

Force in % of body weight.

**Fig. 4:**
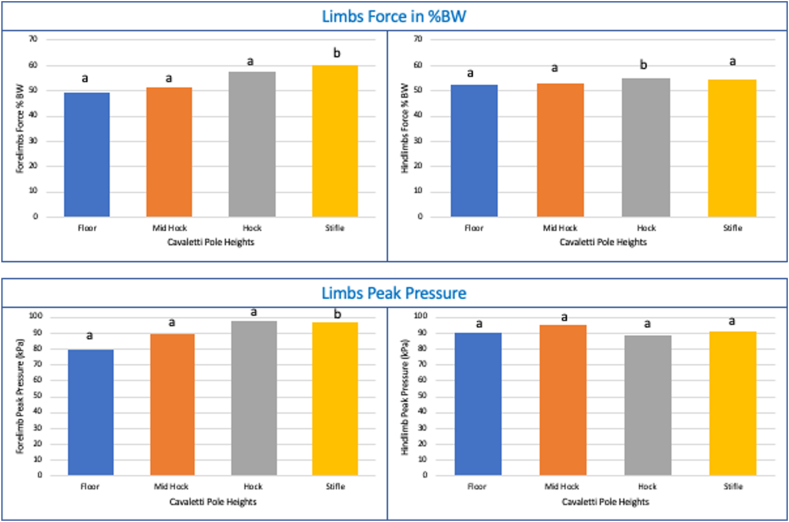
Median scores of forces % BW and peak pressures in individual fore or hindlimb in dogs (n = 8) walking over different poles heights. Different letters indicate significant differences p < 0.05 by Friedman's test.

Force in % of BW showed a statistically significant difference in the forelimbs at different pole heights (χ^2^(3) = 9.375, p < 0.025). Post-hoc analysis revealed statistically significant increase in force % from floor height (*Mdn* = 49.45 %BW) to stifle height (*Mdn* = 60.1 %BW) (*p* = 0.045) (Fig. 4). There was also a significant difference in the hindlimbs (χ^2^(3) = 8.774, p = 0.045), with a significant increase from floor height (*Mdn* = 52.6 %BW) to hock height (*Mdn* = 55 %BW) (*p* = 0.045) (Fig. 4). Furthermore, there was a statistically significant median difference between fore and hindlimb force %BW at stifle level (*Z* = −2.043, *p* = 0.041).

#### 2.1.9 Impulse

Impulse shown no statistically significant difference in the forelimbs at different pole heights (χ^2^(3) = 4.204, p = 0.24). However, there was a significant difference in the hindlimbs (χ^2^(3) = 8.108, p = 0.044), with no significant difference in the hindlimb impulse in the pairwise comparison.

#### 2.1.10 Peak pressures

Peak pressure (kPa) showed a statistically significant difference in the forelimbs at different pole heights, χ^2^(3) = 8.809, p < 0.032. Post-hoc analysis revealed statistically significant increase in peak pressure from floor height (*Mdn* = 79.5 kPa) to stifle height (*Mdn* = 97 kPa) (*p* = 0.045) (Fig. 4). There was no significant difference in the hindlimbs (χ^2^(3) = 0.475, p = 0.924) (Fig. 4).

Only cavaletti at floor level have shown a statistically significant difference between fore and hindlimbs peak pressures (*Z* = −2.173, p = 0.03).

## 3 Discussion

To our knowledge, this is the first study to report the kinetics and joints kinematics of different heights of cavaletti exercise for dogs. The results of our study indicate that the use of cavaletti poles in healthy dogs is significantly beneficial for improving joint ROM, especially at hock height, but may not offer any benefits targeting the range of motion at the hip. It was also evident that kinetics parameters were not significantly changed up to hock height but showed some increases at stifle height. These results are consistent with other equine and canine scientific literature [8,12,18,19], that have investigated kinematics and kinetic changes related to specific therapeutic exercises.

This is the first study to primarily investigate the use of varied heights of cavaletti poles on both the effects of joint ROM and kinetics and are especially useful to veterinary physiotherapists when prescribing exercises, to increase or maintain ROM whilst not increasing concussive forces through the limbs.

Many papers and textbooks will suggest the use of cavaletti poles for certain conditions, however, they fail to provide any justification to suggest why poles should be placed at specific heights, or how it may change the forces placed upon the limbs. regarding impact In horses, it is well known that pole exercises increase ROM without increasing limb load [18], but in horses, this type of exercise is done only at lower heights, such as the low metacarpal level. If cavaletti poles are set higher, this exercise could generate higher impact forces, which is what we have tried to elucidate within our study.

Furthermore, when cavaletti poles were placed at stifle height it was evident that most participants were unable to cope with walking over the poles at this height, and we could observe visually alterations in body position and centre of mass (CoM). Most dogs hesitated and prepared for propulsion to attempt to get over the poles. Although impulse shown did not significantly differ in the forelimbs over the four cavaletti heights (p = 0.24), there was a significant difference in the hindlimbs (p = 0.044). This makes it apparent that canine participants attempting alter their centre of gravity (CoG) and propel themselves over the poles as if jumping [8]. Our results showed lower forces in the hindlimbs than other studies [8], in part because we adjusted the height and distance of the cavaletti exercise relative to individual dog size. Where existing research advises that poles should be raised to stifle height to gain the most flexion and extension of joints [6], we conclude that this can be detrimental to canine joint health and may elicit orthopaedic degenerative changes if this specific exercise is repeated in limbs with pre-existing conditions.

Peak pressures (kPa) in the forelimbs increased from floor height to stifle height (p = 0.045), however here were no significant differences in the hindlimbs (p = 0.924). This could signify that dogs start to shift the weight cranially because of the higher impact on forelimb landing at stifle height. In clinical situations where the dogs have a forelimb injury, increases in forces loaded on limbs during rehabilitation should be introduced with caution, therefore cavaletti exercises at high heights should not be introduced until later in the programme.

The current study found no significant effect on hip joint ROM when poles were placed on the floor versus placement at the mid metatarsals, hock or stifle, in line with existing data [12]Previous work [20] had found that cavaletti poles alter hip ROM, but not extension, confirming the limited benefit of this exercise for hip [5].

All the remaining joints showed an increase in ROM as cavaletti heights increased, most noticeably at hock height. Previous work has described increased flexion (p = 0.01), extension (p = 0.02) ROM (p = 0.01) of the stifle when using poles, which is in line with our results showing an increase in ROM of 47 % [12]. These results are corroborated by the fact that both gait velocity and the number of gait cycles per minute decreases significantly and gait cycle duration increases when compared to a walking gait [10], as it would be expected a decrease in speed and stride frequency when the limbs need to flex more during the exercise.

There are some limitations to our study, which should be acknowledged. The lack of standardisation of speed may be a limiting factor, as this can affect both kinematics and kinetics. We tried to limit its impact by using the same handler throughout our experiment. Another issue relates to skin displacement over bony landmarks in relation to marker placement during movement. Every effort was made to ensure correct marker placement initially, by using the same experienced researcher who was skilled in canine palpation, however to date, no further protocols exist regards adjustment for skin displacement artefact [21].

## 4 Conclusion

Using cavaletti poles at hock level is enough to elicit increase in ROM without increasing forces and pressures, showing it to be an effective and safe exercise. Furthermore, if physiotherapists are wanting to target hip ROM, cavaletti poles may not be the correct exercise to achieve this aim. Having this knowledge will allow veterinary clinicians to be more aware of which cavaletti pole heights are best suited for canine individuals, when deciding on formulating a rehabilitation plan. Lastly, the results of this study indicate that walking over cavalettis at stifle level does not elicit a further increase in ROM and requires substantial adaptions of kinetics and kinematics to permit undisturbed locomotion and may be not suitable to some patients.

## CRediT authorship contribution statement

**Katie Clarke:** Writing – original draft, Investigation, Formal analysis, Data curation, Conceptualization. **Jade Terry:** Supervision, Methodology, Conceptualization. **Scott Blake:** Writing – review & editing, Writing – original draft, Formal analysis. **Roberta Ferro de Godoy:** Writing – review & editing, Writing – original draft, Data curation, Conceptualization.

## Ethics declaration

The data were acquired according to modern ethical standards and was approved by the Animal Welfare and Ethics Committee of Writtle University College before the data was collected. The approval number is 329/2018. The work described has been carried out in accordance with the U.K. Animals (Scientific Procedures) Act, 1986 and associated guidelines. A written informed consent was obtained from the owners of the participants of the study before the collection of data from the dogs. Veterinary consent was also required to discount any current or underlying orthopaedic conditions that could hinder results or compromise the animal's welfare.

## Data and code availability

Data will be made available on request.

## Funding

This research did not receive any specific grant from funding agencies in the public, commercial, or not-for-profit sectors.

## Declaration of competing interest

The authors declare that they have no known competing financial interests or personal relationships that could have appeared to influence the work reported in this paper.

## References

[1] Shaw K.K. (2017). Physical rehabilitation for canine patients post cranial cruciate ligament surgery. Companion Anim.

[2] Millis D., Levine D. (2014).

[3] McGowan C.M., Cottriall S. (2016). Introduction to equine physical therapy and rehabilitation. Vet. Clin. N. Am. Equine Pract..

[4] Carver D. (2015).

[5] Davidson J.R., Kerwin S.C., Millis D.L. (2005). Rehabilitation for the orthopedic patient. Vet. Clin. Small Anim. Pract..

[6] Levine D., Millis D.L., Marcellin-Little D.J. (2005). Introduction to veterinary physical rehabilitation. Vet. Clin. Small Anim. Pract..

[7] Prydie D., Hewitt I. (2015).

[8] Charalambous D., Strasser T., Tichy A., Bockstahler B. (2022). Ground reaction forces and center of pressure within the paws when stepping over obstacles in dogs. Animals.

[9] Saunders D.G., Walker J.R., Levine D., Millis D., Levine D. (2014). Canine Rehabilitation and Physical Therapy.

[10] Blake C.A., Looney A.L., Merrill T.D. (2024). The impact of cavaletti height on dogs' walking speed and its implications for ground reaction forces. Front. Vet. Sci..

[11] Arifin W.N., Zahiruddin W.M. (2017). Sample size calculation in animal studies using resource equation approach. Malays. J. Med. Sci..

[12] Holler P.J., Brazda V., Dal-Bianco B., Lewy E., Mueller M.C., Peham C., Bockstahler B.A. (2010). Kinematic motion analysis of the joints of the forelimbs and hind limbs of dogs during walking exercise regimens. Am. J. Vet. Res..

[13] O'Sullivan K., Murray E., Sainsbury D. (2009). The effect of warm-up, static stretching and dynamic stretching on hamstring flexibility in previously injured subjects. BMC Muscoskel. Disord..

[14] Blake S., de Godoy R.F. (2021). Kinematics and kinetics of dogs completing jump and A-frame exercises. Comp Exerc Physiol.

[15] Klinhom S., Chaichit T., Nganvongpanit K. (2015). A comparative study of range of motion of forelimb and hind limb in walk pattern and trot pattern of Chihuahua dogs affected and non-affected with Patellar Luxation. Asian J. Anim. Vet. Adv..

[16] Böddeker J., Drüen S., Meyer-Lindenberg A., Fehr M., Nolte I., Wefstaedt P. (2012). Computer-assisted gait analysis of the dog: comparison of two surgical techniques for the ruptured cranial cruciate ligament. Vet. Comp. Orthop. Traumatol..

[17] Keebaugh A.E., Redman-Bentley D., Griffon D.J. (2015). Influence of leash side and handlers on pressure mat analysis of gait characteristics in small-breed dogs. J. Am. Vet. Med. Assoc..

[18] Clayton H.M., Stubbs N.C., Lavagnino M. (2015). Stance phase kinematics and kinetics of horses trotting over poles. Equine Vet. J..

[19] Walker V.A., Tranquillle C.A., MacKechnie-Guire R., Spear J., Newton R., Murray R.C. (2022). Effect of ground and raised Poles on kinematics of the walk. J. Equine Vet. Sci..

[20] Headrick J., Hicks D., McEachern G. (2006). 2nd World Veterinary Orthopedic Congress Veterinary Orthopedic Society.

[21] Lin C.C., Chang C.L., Lu M., Lu T.W., Wu C.H. (2018). Quantification of three-dimensional soft tissue artifacts in the canine hindlimb during passive stifle motion. BMC Vet. Res..

